# The influence of the land-to-sea macroevolutionary transition on vertebral column disparification in Pinnipedia

**DOI:** 10.1098/rspb.2023.2752

**Published:** 2024-04-10

**Authors:** Juan Miguel Esteban, Alberto Martín-Serra, Alejandro Pérez-Ramos, Natalia Rybczynski, Katrina Jones, Borja Figueirido

**Affiliations:** ^1^ Departamento de Ecología y Geología, Facultad de Ciencias, Universidad de Málaga, Campus Universitario de Teatinos s/n, 29071, Málaga, Spain; ^2^ Department of Palaeobiology, Canadian Museum of Nature, Ottawa, ON, Canada K1P 6P4; ^3^ Department of Earth Sciences & Department of Biology, Carleton University, 1125 Colonel By Drive, Ottawa, ON, Canada K1S 5B6; ^4^ Department of Earth and Environmental Sciences, University of Manchester, Williamson Building, Oxford Road, Manchester M13 9PL, UK

**Keywords:** vertebral column, evolution, pinnipedia, disparity

## Abstract

The repeated returns of vertebrates to the marine ecosystems since the Triassic serve as an evolutionary model to understand macroevolutionary change. Here we investigate the effects of the land-to-sea transition on disparity and constraint of the vertebral column in aquatic carnivorans (Carnivora; Pinnipedia) to assess how their functional diversity and evolutionary innovations influenced major radiations of crown pinnipeds. We use three-dimensional geometric morphometrics and multivariate analysis for high-dimensional data under a phylogenetic framework to quantify vertebral size and shape in living and extinct pinnipeds. Our analysis demonstrates an important shift in vertebral column evolution by 10–12 million years ago, from an unconstrained to a constrained evolutionary scenario, a point of time that coincides with the major radiation of crown pinnipeds. Moreover, we also demonstrate that the axial skeleton of phocids and otariids followed a different path of morphological evolution that was probably driven by their specialized locomotor strategies. Despite this, we found a significant effect of habitat preference (coastal versus pelagic) on vertebral morphology of crown taxa regardless of the family they belong. In summary, our analysis provides insights into how the land-to-sea transition influenced the complex evolutionary history of pinniped vertebral morphology.

## 1. Introduction

The vertebral column of tetrapods (land-dwelling vertebrates) is a complex, regionalized and multifaceted structure composed of several articulated subunits (vertebrae). It plays a pivotal role in the protection of the spinal cord, locomotion, and body support (e.g. [1]). Therefore, understanding patterns of vertebral evolution is fundamental for understanding the evolution of the tetrapod body plan [2].

The ecological demands of different environments can influence vertebral morphology (e.g. [3–13]). In terrestrial taxa, the stiffness and stability of the spine can support body weight and transmit propulsive forces from the limbs [14] but the axial skeleton of secondarily aquatic tetrapods maintains buoyancy in water and vertebral morphology is strongly influenced by swimming adaptations (e.g. [4,8,15]). Therefore, the marine environment releases (in part) the axial skeleton from its role in body support under gravity [4,16–19], and it allows oscillatory or undulatory movements [20]. On the other hand, this new physical environment imposed selective regimes leading to a major reorganization of their body plans, including the acquisition of streamlined, torpedo-shaped bodies, the transformation of limbs into flippers, and sometimes the appearance of caudal flukes [21–23]. Therefore, tetrapods that are secondarily adapted to the marine realm experienced a radical transformation of their axial skeletons according to the new locomotory demands of the aquatic environment [21–23] and the release from other physical aspects of the terrestrial one [4]. Despite this, few studies (e.g. [4,8,15]) have quantified the impact of the land-to-sea evolutionary transition on the evolution of the vertebral column in taxa that transitioned from land to sea and neither of them has assessed changes of axial disparity *sensu* Figueirido *et al*. [10]. Morphological disparity is crucial to understand how the ecological roles and functional diversity evolve and how the appearance of key innovations allow to cope with new ecological opportunities, which ultimately influences the evolutionary success of certain lineages [24].

**Figure 1.**
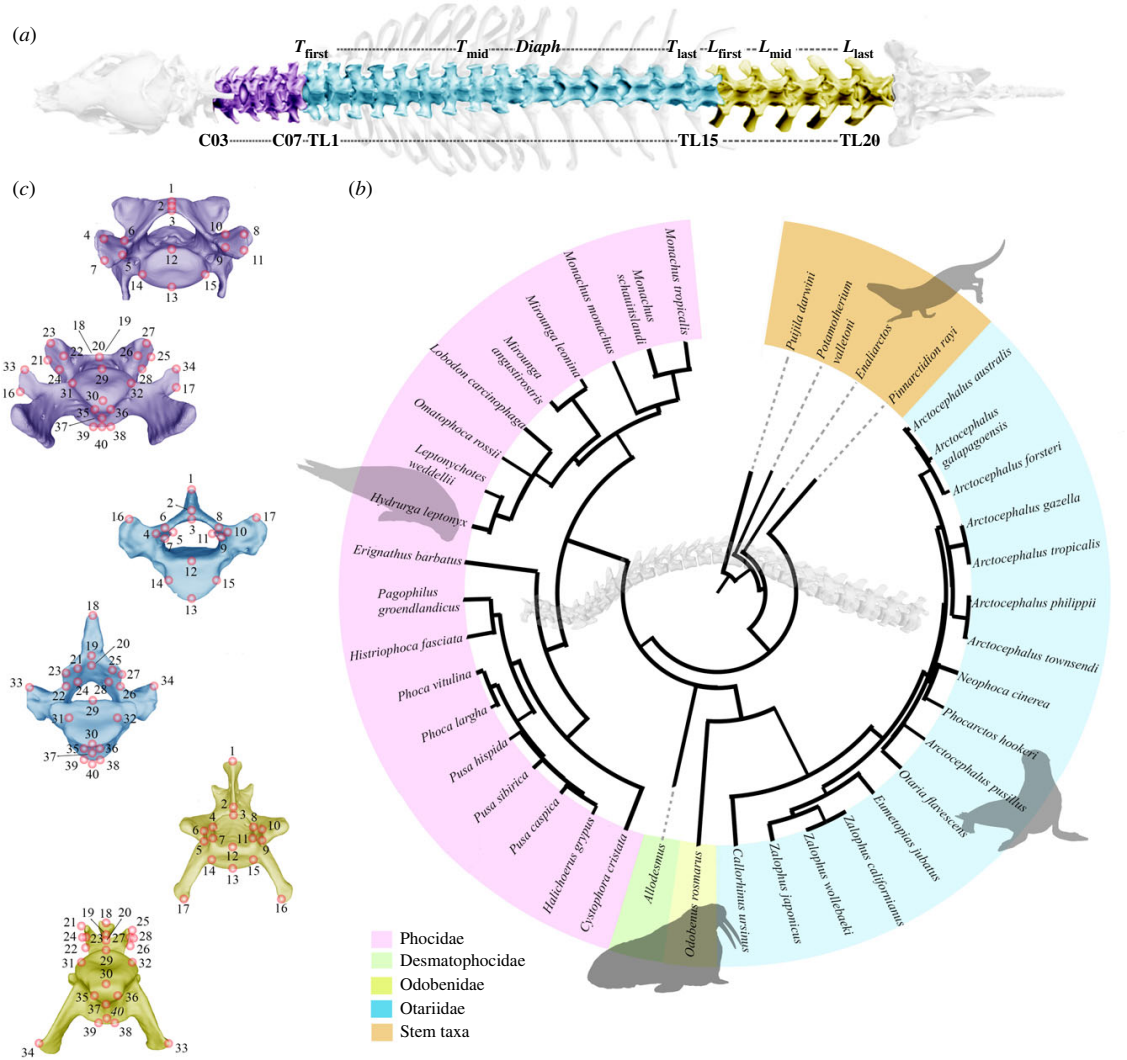
Vertebral column analysis of pinnipeds. (*a*) Sampling schemes used in this paper with different hypotheses of homology. (*b*) Landmarks digitized on the pinniped vertebrae (electronic supplementary material, table S2). From top to down: cervical vertebrae in cranial and caudal views, thoracic vertebra in cranial and caudal views, and lumbar vertebrae in dorsal and ventral views. Vertebrae belong to a *Zalophus californianus* as an example. (*c*) Phylogenetic hypothesis used in this paper for comparative analyses (see text for details).

In this study, we quantify disparity and constraint of the vertebral column (figure 1*a*) in crown and stem pinnipeds (Carnivora; Pinnipedia) *sensu* Figueirido *et al*. [10] to explore both local scale (within regions) and broad scale (between regions) morphological variabilities. Pinnipeds (seals and kin) are a group of marine carnivoran mammals (figure 1*b*) that have evolved a range of specialized adaptations to inhabit the aquatic environment, including a streamlined shape, reduced external ear pinnae, paddle-like limbs, reduced tail, and the genitals and mammary glands retracted beneath the skin (e.g. [20,25]). The evolution of the vertebral column of pinnipeds is interesting because crown groups exhibit different swimming styles (i.e. pectoral rowing of otariids, pelvic oscillation of phocids and a mixed type of locomotion of odobenids). Therefore, they offer the possibility to investigate shared changes in their axial skeleton due to their colonization of the marine realm and distinctive changes related to their different swimming styles. Moreover, pinnipeds still have some capacity to move on land, where they perform some important activities, such as mating and giving birth [26]. Accordingly, unlike other more specialized marine mammals such as cetaceans, they are fully regionalized including a sacral region and they exhibit pre- and postzygapophyses [27]. However, whether they experienced a change in disparity from their close terrestrial relatives through their evolutionary history is unknown.

Our main goal is to investigate how the release of the axial skeleton from its role in body support under gravity impacts on axial disparity in a group of marine mammals that still retain some capacity to move on land, and whether the release of this gravitational constraint have allowed changes in disparity in relation with the different ecologies exhibited today by crown taxa. Specifically, in the present study: (i) we quantify vertebral morphology in crown and stem pinnipeds to explore potential differences or similarities between crown groups and between stem and crown taxa; (ii) we explore patterns of morphological disparity and constraint to test whether the evolution of the vertebral column of pinnipeds has been constrained or unconstrained across time; and (iii) we quantify the influence of ecology on vertebral morphology to investigate whether the selective regimes imposed by the marine environment have modified vertebral morphologies to accomplish the new functional demands.

## 2. Material and methods

In this study, we analysed 1099 presacral vertebrae, from the third cervical vertebra to the last lumbar, belonging to 49 specimens (electronic supplementary material, table S1; figure 1*a*). These specimens belong to 28 species of extant pinnipeds (Phocidae: 28 specimens/16 species; Otariidae: 14 specimens/11 species; Odobenidae: 2 specimens of *Odobenus rosmarus*) plus four stem pinnipeds, *Potamotherium valletoni* [28], *Puijila darwini* [29], *Pinnarctidion rayi* [30] and *Enaliarctos* indet*.* [31] (figure 1*b*). We also included one desmatophocid, *Allodesmus kelloggi* [32] (figure 1*b*; electronic supplementary material, table S1).

We scanned each vertebra with a surface scanner EinScan Pro 2X Plus (resolution 0.04 mm) and we obtained a three-dimensional mesh for each vertebra. We repaired the models using the Geomagic Essentials software [33] with the tool ‘polygons: fill holes'. Then, we smoothed the mesh and reduced the resolution without losing morphological topology for subsequent analysis. In those few vertebrae with incomplete parts, they were reconstructed using the ‘mirroring’ algorithm from the mid-sagittal plane.

A series of 40 homologous, three-dimensional landmarks were digitized in all vertebrae to capture their main morphological features (figure 1*c*; electronic supplementary material, table S2), using the software *Stratovan Checkpoint* [34]. These landmarks were chosen to capture morphological variation in muscle attachments and joint areas and were digitized by one of us (JME). The raw landmarks coordinates were imported as text format into R environment [35].

A phylogeny for the species included in the sample (figure 1*b*) was assembled using Mesquite [36] and the *ape* package [37]. We used the topology and branch lengths for living species of the supertree published by Nyakatura and Bininda-Edmonds [38], because it is the most recent complete phylogeny for this mammalian order (but see [39]). The extinct species collected here (i.e. *Po. valletoni,* *Pu. darwini, Pi. rayi, A. kelloggi* and *Enaliarctos* sp.) were assembled using the phylogeny of Paterson *et al*. [40]. Therefore, we considered Otariidae and Odobenidae (Otarioidea) to be more closely related than either is to Phocidae. The extinct family Desmatophocidae was considered as closely related to Otarioidea following the results of Paterson *et al*. [40]. Nevertheless, some debate exists regarding the phylogenetic relationships at various levels within Paterson's topology, including the affinities of desmatophocids or the relationships of pinnipeds with other arctoid groups (e.g. [41,42]). Despite this, we used this topology because it was obtained through a Bayesian analysis employing ‘tip-dating’ of a combined molecular-morphological dataset (i.e. the total evidence).

We performed a Procrustes superimposition [43] for each region of the vertebral column (cervical, thoracic, and lumbar) using the *geomorph* package of R [44]. The asymmetric component of shape variation was removed and, therefore, only symmetric variations were analysed. Those species represented by more than one specimen were averaged by vertebra.

### (a) Quantifying vertebral morphology in crown and stem pinnipeds

We performed a principal components analyses (PCA) for each region, and we computed phenotypic trajectory analysis (PTA) [45,46] by vertebral position with *geomorph* [44]. These trajectories were visualized in the morphospace depicted from the first two eigenvectors for each region. Our primary objective is to quantitatively examine whether phocids and otariids (other groups are only represented by one or two species/specimens) exhibit variations in the morphological patterns identified in the morphospaces defined by the first two eigenvectors obtained in the PCA performed from each column region.

### (b) Investigating phenotypic disparity and constraint across time

We computed the Procrustes variance for each vertebra as a proxy for morphological disparity, using *geomorph* [44]. We controlled for some biases (i.e. different number of species of phocids and otariids and the presence of outliers) performing the sum of variance and the sum of ranges for each vertebra. We computed a bootstrapping procedure to provide a distribution of values for each using the *disRity* package of R [47].

We also performed disparity-through time analyses (DTT) [48] for each vertebra and we also computed the morphological disparity index (MDI) as a measure of morphospace partitioning with the *geiger* package of R [49]. Our primary objective here is to test whether the evolution of the vertebral column of pinnipeds follows a constrained or unconstrained evolutionary scenario across time.

As there are pinniped species with slightly different thoracic and lumbar counts, the homology between vertebrae may be problematic (see [10] for details). For this reason, we repeated these analyses for a set of selected vertebrae with an alternative hypothesis of homology that ensures comparable results using the ‘selected vertebrae approach’ [10]: first, middle and last thoracics, the diaphragmatic vertebra, and first, middle and last lumbars. The analyses of disparity by position and using the 'selected vertebra approach’ were repeated for a sample of phocids and otariids separately.

### (c) The influence of habitat preferences on vertebral morphology in crown pinnipeds

The presence of an association between ecology and vertebral morphology was tested for extant pinnipeds. As the locomotor mode is highly associated with phylogeny [20,50], we tested the association between vertebral morphology and the preferred habitat of the species under study (i.e. coastal versus pelagic), because we have representatives of both ecological groups within phocids and otariids. We classified as pelagic all species that, at least in some periods of the year, can cross long distances of open ocean, even though they may inhabit territory near the coast for most of their lifetimes. The remaining ones (those that are usually confined to near-shore habitats) have been classified as coastal (electronic supplementary material, table S3).

We performed a phylogenetic ANOVA (or PGLS) for each vertebra according to their position and for each of ‘the selected vertebrae’ according to anatomical criteria to cover both hypotheses of homology using *geomorph* [44]. In cases where vertebral shape is associated with species ecology, we modelled the hypothetical vertebral morphologies for both coastal and pelagic species using topological deviations of shape (see Figueirido *et al*. [10] for further details).

## 3. Results and discussion

### (a) Phenotypic variability by region and axial divergence in crown pinnipeds

#### (i) Cervical region

**Figure 2.**
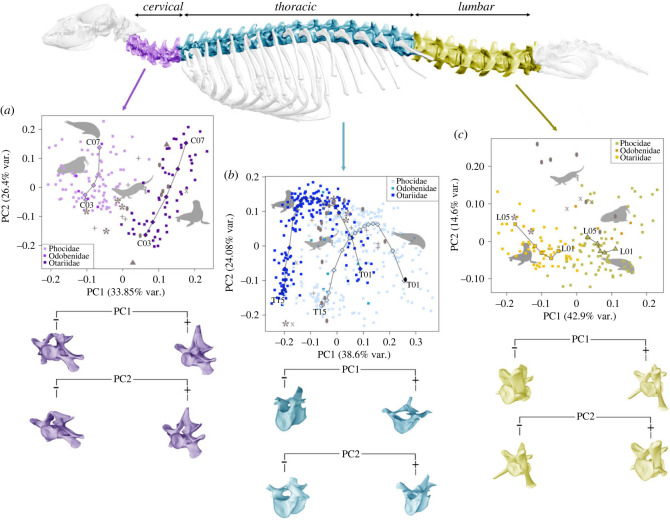
Morphospaces derived from the PCAs of vertebral shape. (*a*) PC1 versus PC2 for the cervical region. (*b*) PC1 versus PC2 for the thoracic region. (*c*) PC1 versus PC2 for the lumbar region. The 3D models correspond to morphological changes associated with the extreme scores on the first two eigenvectors. Curves within morphospaces represent the average shape of each vertebra (only phocids and otarids). Symbols for fossil taxa are as follows: asterisks, *Pu. darwini*; crosses, *Po. valletoni*; ovals, *A. kelloggi*; triangles, *Enaliarctos* sp.; X, *Pi. rayi*.

The results obtained from the PCA for the cervical region yielded two first principal components (PCs) that jointly account for more than 60% of original variance. PC1 shows that otariids score positively on this eigenvector and phocids plus *Odobenus* score negatively. The extinct species *Po. valletoni* and *Pu. darwini* have intermediate values but *Pi. rayi*, *Allodesmus* and *Enaliarctos* have similar scores than otariids (figure 2*a*). The morphological features associated with the first eigenvector are the shape of the vertebral body (cylindrical or disk-shaped) and the length of the spinous process: the cervical vertebrae of otariids have cylindrical bodies and long spinous processes, whereas the ones of phocids plus *Odobenus* are disk-shaped and they have short spinous processes (figure 2*a*). The relationship of these morphological changes with development of different muscles are described in the electronic supplementary material (figure S1).

The second PC shows differences between vertebral positions from C03s to C07s (figure 2*a*). The main morphological differences accounted for by this eigenvector are the length of the spinous and transverse processes: anterior vertebrae have shorter spinous processes as well as longer transverse processes than posterior vertebrae (figure 2*a*). The relationship of these morphological changes with the degree of development of different muscles are described in the electronic supplementary material (figure S1).

Vertebral shape is a good indicator of intervertebral joint flexibility or rigidity (i.e. [15]), and the processes play a crucial role in the movement of the whole spine [15]. Indeed, some studies (e.g. [51,52]) have demonstrated a robust correlation between the intervertebral motion inferred from morphometric versus cadaveric data, especially the shape and dimensions of zygapophysial articular facets (e.g. [51]).

A spinous process with a large dorsal projection (or height) indicates the presence of a well-developed muscular mass, increasing passive stiffness in the sagittal plane [2,53]. The orientation of the spinous process indicates whether stability is more important than strength or vice versa; if it is cranially oriented indicates that strength is prioritized over stability, whereas if it is caudally oriented, stability is prioritized over strength [2,15]. Additionally, transverse processes act as elevators of epaxial muscles for lateral movements [2,54]; in general, a larger transverse process indicates greater power in lateral movements but also increases spinal stiffness due to the presence of more muscular mass [2,15]. The size of the vertebral body has a considerable impact on the degree of passive flexibility of intervertebral joints. Vertebrae with a short body or disk-shaped increase stiffness, while those with large bodies or spool-shaped increase intervertebral flexibility [2,15].

Therefore, according to the PCA results, the cervical vertebrae of phocids (and odobenids) exhibit a morphology that indicates a stiff neck with reduced muscle mass [55]. By contrast, the cervicals of otariids reflect a high degree of neck flexibility with well-developed neck muscles [55] (electronic supplementary material, figure S1). This indicates that vertebral morphology reflects how otariids and phocids solve the problem of drag at top speeds. Keller [55] proposed that phocids maintain a passive rigid neck but otariids maintain neck rigidity by muscular contraction. On the other hand, the cervicals of odobenids are like that of phocids, which may indicate that they maintain a passive rigid neck to forage small invertebrates living on the seafloor, for fighting, or to haul themselves out of the water and onto the sea ice, but not to manage drag at top speeds [26].

The extinct species sampled, *A. kelloggi*, *Enaliarctos* and *Pi. rayi* plot within the otariids, while *Pu. darwini* and *Po. valletoni* fall between otariids and the combination of phocids plus odobenids (figure 2*a*). This suggests that *A. kelloggi*, *Enaliarctos* and *Pi. rayi* would maintain greater mobility in the cervical region with well-developed muscles of the neck. On the other hand, *Pu. darwini* and *Po. valletoni* present less mobility than otariids but more than phocids plus odobenids.

Our results obtained for the cervical region may suggest that the neck of crown pinnipeds evolved from the intermediate morphologies characteristic of stem taxa (figure 2*a*) into two different solutions to cope with the aquatic demands: (i) in otariids, the desmatophocid *A. kelloggi*, and some stem taxa such as *Enaliarctos* and *Pi. rayi*, with cervical morphologies prevailing flexibility instead of stiffness; and (ii) in extant phocids and odobenids which exhibit a more derived morphology to maintain passively a stiff neck with disk-shaped vertebrae.

Our results contrast with those obtained by other authors for the cervico-thoracic series in semiaquatic crocodylians [56], as this region exhibited conservative patterns—although two sub-regions within the crocodylian neck were differentiated.

The trajectory analysis indicates that both phocids and otariids show significant differences in path length, which suggests that the amount of change between the first and last cervical is different for both groups (table 1). Therefore, phocids present less morphological heterogeneity across C03–C07 than otariids. Additionally, both families also exhibit significant differences in trajectory direction, which indicates that changes from C03 to C07 are different in each group. This confirms that both families evolved towards two different solutions to adapt the cervical region to the aquatic environment, and the morphological changes are not uniform along the region, which suggests more complex modifications in the development of this region in both groups.

**Table 1.** Results of the phenotypic trajectory analyses performed for the cervical, thoracic and lumbar regions. Only the *p*-values (significant ones in bold) to test for differences in PTA between phocids and otariids are shown.

	cervical	thoracic	lumbar
path length	**0**.**001**	0.994	0.131
trajectory direction	**0**.**046**	**0**.**001**	0.232
trajectory shape	0.44	0.841	0.806

#### (ii) Thoracic region

The results obtained from the PCA of the thoracic region yielded two first PCs that sum approximately 63% of the original variance. The morphospace depicted by these two PCs show a ‘horseshoe’ distribution of vertebrae, with the extreme vertebrae (T01 and T16) with opposite scores in PC1 but with negative scores in PC2, whereas the central vertebrae (≈T05–T08) possess the most positive scores in PC2 (figure 2*b*). Vertebrae with positive scores on the first PC have disk-shaped bodies and extended transverse processes, whereas those with negative ones have cylindrical bodies and short transverse processes, among other traits. On the second PC, vertebrae scoring negatively have wider bodies and shorter spinous processes, whereas those scoring positively have taller bodies and longer spinous processes, among other traits (figure 2*b*). The relationship of these morphological changes with the degree of development of different muscles are described in the electronic supplementary material (figure S2).

The thoracic series of pinnipeds is characterized by having disk-shaped anterior vertebrae and more cylindrical vertebrae in posterior portion of the trunk (figure 2*b*). Despite this, phocids and otariids exhibit different distribution of vertebrae evidenced by two horseshoes that are clearly discernible in the thoracic morphospace of figure 2*b*. As in the cervical series, trunk vertebrae of phocids are characterized by short vertebral bodies and elongated transverse processes but trunk vertebrae of otariids are cylindrical with long spinous processes. Therefore, the thoracic vertebrae of phocids probably reflect a stiffer trunk, in response to pelvic oscillation, a swimming mode that necessitates a rigid core in the thoracic region to generate powerful thrust with the lumbar series [15]. However, the thoracic vertebrae of otariids probably reflects their flexible trunk, which is essential for the ability of manoeuvring and turning [15] (electronic supplementary material, figure S2). On the other hand, the living walrus seem to display an intermediate trajectory of vertebral morphotypes between the trajectories exhibited for otariids and phocids.

The included stem taxa (*Pu. darwini, Po. valletoni, A. kelloggi*) exhibit intermediate vertebral morphotypes to the trajectories described by crown pinnipeds. We hypothesize that stem taxa exhibit thoracic regions with intermediate levels of flexibility and stiffness to that of living otariids and phocids, respectively.

The trajectory analysis of the thoracic region reveals that both phocids and otariids do not show significant differences in path length, which indicates that both families have a similar heterogeneity across the thoracic series (table 1). However, trajectory direction is significantly different for both families, suggesting that vertebral morphology across the thoracic series varies phenotypically towards different morphotypes (i.e. both groups follow different paths in terms of morphological variation across the thoracic series). We interpret that these differences observed between crown pinnipeds are related to the fact that phocids probably present a trade-off between stiffness and movement in their most-posterior thoracic series but the spine of otariids does not present this trade-off because it does not actively participate in generating thrust during swimming.

#### (iii) Lumbar region

The results obtained from the PCA of the lumbar region yielded two first PCs that account for ≈ 55% of total variance. PC1 shows differences between taxonomic groups. Otariids have negative scores, whereas phocids and *Odobenus* have positive ones (figure 2*c*). The main morphological features accounted for by the first PC are the shape of the vertebral body and the length of the transverse processes (figure 2*c*). Positive scores indicate disk-shaped vertebral bodies and long transverse processes. By contrast, negative scores indicate cylindrical vertebral bodies and short transverse processes (figure 2*c*). PC2 shows differences between species and the position of each vertebra with a morphological change associated with the length of the spinous and transverse processes; while positive scores indicate short spinous and transverse processes, negative scores indicate long processes (figure 2*c*). The relationship of these morphological changes with the degree of development of different muscles are described in the electronic supplementary material (figure S3).

In this morphospace, phocids and odobenids exhibit elongated transverse processes, suggesting a higher degree of mobility in the horizontal plane, which correlates with their mode of locomotion [50]. This implies a greater muscle mass associated with these transverse processes, albeit accompanied by a slight reduction in sagittal plane flexibility [50]. Conversely, otariids display reduced vertebral processes in this region while retaining their spool-shaped vertebrae, indicating a higher level of lumbar flexibility compared to phocids (electronic supplementary material, figure S3).

It is hypothesized that *Po. valletoni* had terrestrial habits like those of a modern otter [28]. Indeed, the brain endocast of *Po. valetoni* reveals an enlarged coronal gyrus, providing additional evidence that it was a whisker specialist like modern pinnipeds, and it did not use their hands for foraging as living fissipeds do [57]. Strikingly, the lumbar vertebrae of *Po. valletoni* cluster with the lumbar vertebrae of otariids. This may suggest that the lumbar morphology found in otariids is the one that supports such terrestrial activity, as they move using all fours when locomoting on land, while phocids only use the axial skeleton [50]. On the other hand, the stem pinniped *Pi. rayi* shows the opposite pattern (i.e. with a lumbar morphology typical of phocids).

The locomotor mode of *Allodesmus* is contentious because Berta & Adam [58] proposed that this taxon probably used pelvic oscillation like phocids, but Bebej [59] proposed that *Allodesmus* used forelimb-swimming like otariids. However, the flexible thoracic and short lumbar region suggest that it probably deployed a combination of fore- and hindlimb movements. Other studies have also suggested that *Allodesmus* possessed several features consistent with forelimb propulsion, but also displays adaptations for hindlimb swimming [15,60].

Our results indicate that *A. kelloggi* exhibits a unique combination of axial morphology because the first lumbar vertebra clusters with phocids, but the remaining lumbars show extreme values in PC2, indicating that its posterior lumbars are characterized by very short transverse processes but large zygapophyseal widths. In functional terms, this may indicate that this desmatophocid, which still retains seven lumbar vertebrae (unlike crown pinnipeds), does not possess a lumbar region with the same strength of phocids to move solely with the posterior part of the spine (figure 2*c*). Therefore, we hypothesize that *A. kelloggi* employed a mixed type of locomotion, or that the thrust would not be produced entirely from the lumbar region. Instead, only the first lumbar vertebra was likely involved in exerting propulsive forces and a recent study revealed that the sacrum of *Allodesmus* was more like that of phocids than to that of otariids [16].

Phocids and otariids had similar trajectories based on PTA (table 1), indicating that both groups follow similar directions and maintain similar shapes, differing primarily in terms of their transverse processes (figure 2*c*). The lumbar region exhibits high variability (disparity) in vertebral positions, both within each species and across various groups. Substantial dispersion exists even among species within the same family, and these differences are further amplified when comparing different families. Following our results, we hypothesize that the lumbar region is the one that presents more phenotypic variability, most probably because it is the one most influenced by locomotor adaptation on both land and water.

### (b) Phenotypic disparity, disparity through time curves and constrained evolution

**Figure 3.**
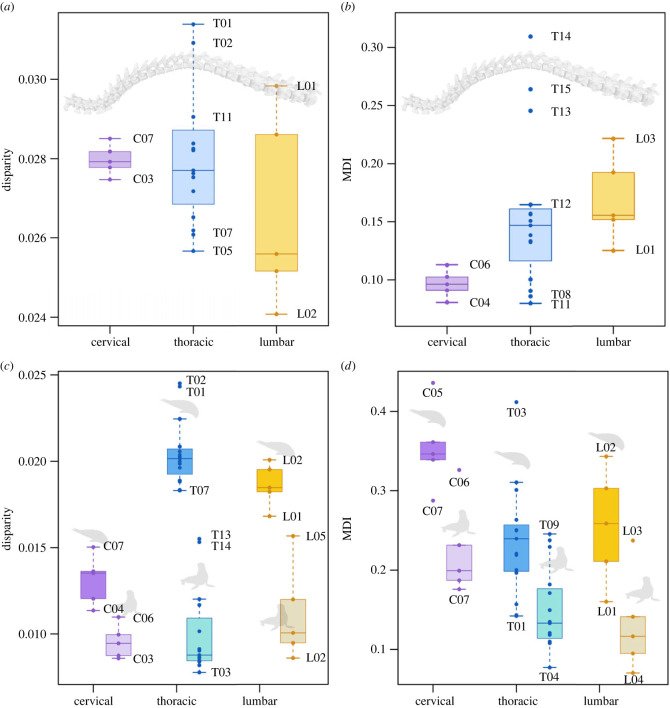
Results for the analyses of disparity and MDI according to vertebral position. (*a*) Box plots of disparity values for the whole sample of pinnipeds for each morphological region. (*b*) Box plots of MDI values (as proxy for constraint) for the whole sample of pinnipeds for each morphological region. (*c*) Box plots of disparity values for otariids and phocids in each morphological region; (*d*) Box plots of MDI values for otariids and phocids in each morphological region. The boxplot shows the median (horizontal line), the 25–75 quartiles (coloured box) and the most extreme values within 1.5 times the interquartile range (whiskers). For the results using the selected vertebrae approach see electronic supplementary material, table S5.

Fissipeds display an increasing disparity trend from the thoracic to the last lumbar [10], but disparity in pinnipeds is concentrated in vertebrae located at interregional boundaries (mainly T01, T02, T11, L01, L02) and cervical vertebrae possess similar disparity values to other thoracic vertebrae (figure 3*a*; electronic supplementary material, figure S4*a* and table S4).

The results obtained for sum of variances and ranges of disparity show very similar patterns. Moreover, the comparison of the bootstrap median values with the observed ones (electronic supplementary material, figure S5; electronic supplementary material, tables S5–S8) indicates that they are not influenced by sampling biases.

**Figure 4.**
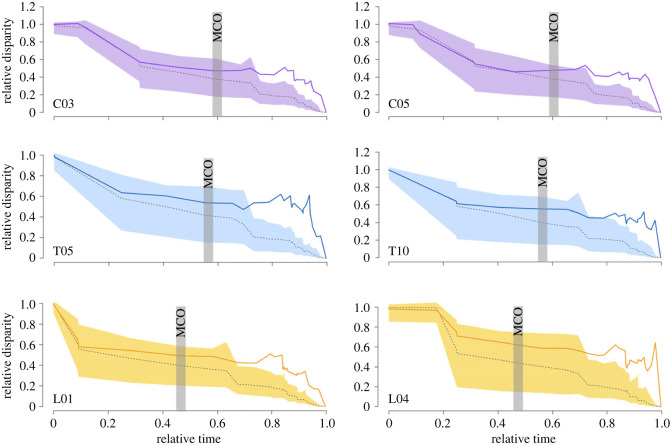
Disparity-through-time (DTT) plots from the results obtained for all pinnipeds. Only a pair of vertebrae per region are shown for clarity (see electronic supplementary material, figure S5 for all vertebrae and electronic supplementary material, figure S6 for the results obtained from the selected vertebrae approach). Relative time values have been used, with the value of 0.0 representing the root of the phylogeny and 1.0 representing the present. The solid line shows the mean DTT from the empirical dataset, while the dashed line represents the results of the simulated datasets. Shaded areas indicate the 95% DTT range for the simulated data under Brownian motion. MCO: Miocene Climatic Optimum.

All pinniped vertebrae exhibit very low MDI values (figure 3*b*; electronic supplementary material, figure S4*b* and table S4), ranging from 0.061 (in T11) to 0.365 (in T14) and disparity through time curves consistently show a low proportional subclade disparity through most of the evolutionary history of Pinnipedia (figure 4; electronic supplementary material, figures S6–S10). L03 exhibit the highest MDI values among the lumbar series, followed by the last thoracics (T13–T15) and the cervicals and mid thoracics (T08–T12) exhibit low values (figure 3*b*; electronic supplementary material, figure S4*b* and table S4). Therefore, pinniped subclades have individually low disparities compared to the total clade, like those expected under random evolution (Brownian motion), suggesting partitioning of morphospace among subclades and symptomatic of unconstrained evolution. Again, these results contrast substantially with those obtained for fissipeds by Figueirido *et al*. [10] in which high MDI values are evident for vertebral evolution (MDI ranging from 0.24 to 0.47) and disparity through time curves consistently show a high proportional subclade disparity through most of the evolutionary history of fissipeds, which indicates a constrained evolution (DTT curves depart from the confidence interval for the expected evolution under Brownian motion).

The pattern of unconstrained evolution obtained for pinnipeds is maintained for most their evolutionary history, until approximately 10–12 million years ago (0.6–0.8 relative time; figure 4; electronic supplementary material, figures S6–S10). From this point of time, disparity through time curves consistently depart from the confidence interval of Brownian evolution and the vertebral column of pinnipeds is characterized by a constrained evolutionary scenario in which subclades occupy a large proportion of the whole-clade space. This suggests that all clades recurrently occupy the same morphospace regions. Strikingly, these results coincide with those obtained for the evolution of the pinniped skull in which a shift in cranial shape occurred by a release from evolutionary constraint without increasing the rate of evolution until recently [61]. Therefore, our results indicate that these changes in the pinniped body plan occurred after the Miocene Climatic optimum (14–16 Ma [62]). This peak in temperature of the middle Miocene marked an important increase of ocean productivity, which probably increased marine species richness [63–65]. Moreover, the origination of the modern-style diversity gradient arose 15 million years ago, a factor that may have favored niche partitioning among taxa and more opportunities for speciation at low latitudes [66].

Vertebral disparities and MDI values computed separately for phocids and otariids indicate that phocids show higher disparity values than otariids and the pattern of disparity along the spine is also different for each group (figure 3*c,d*; electronic supplementary material, figures S8–S10 and table S4). Phocids show the lowest disparity values for the cervicals, while their thoracics and lumbars are substantially more disparate. On the other hand, the otariids present similar disparity values in all regions (figure 3*c*; electronic supplementary material, figure S4*a*). Strikingly, the MDI values are higher for phocids than for otariids (figure 3*d*; electronic supplementary material, figure S4*b*). This indicates that despite the higher disparity of phocids relative to the one of otariids, different subclades of phocids recurrently occupy the same portion of the morphospace (electronic supplementary material, figures S5–S10 and table S4) and, therefore, a slightly more constrained evolution for their axial skeleton. We hypothesize that these differences in disparity and constraint between phocids and otariids relate to their different locomotor adaptations in water and the role of the vertebral column on it. In phocids, the vertebral column is involved in generating thrust and, therefore, it may be more strongly influenced by selection, which usually promotes changes (higher disparity), but only adaptive morphologies can be achieved (higher constraint). In contrast, as otariids use their fore flippers to generate thrust and their spine is only involved in manoeuvring and turning [15,50], the influence of natural selection on the whole vertebral column may be low, making morphological variation similar to that expected by chance (lower but more unconstrained disparity). In any case, our results clearly suggest that pinnipeds have undergone two different pathways in response to the challenges imposed by the marine environment.

### (c) Ecological signal per vertebra

**Figure 5.**
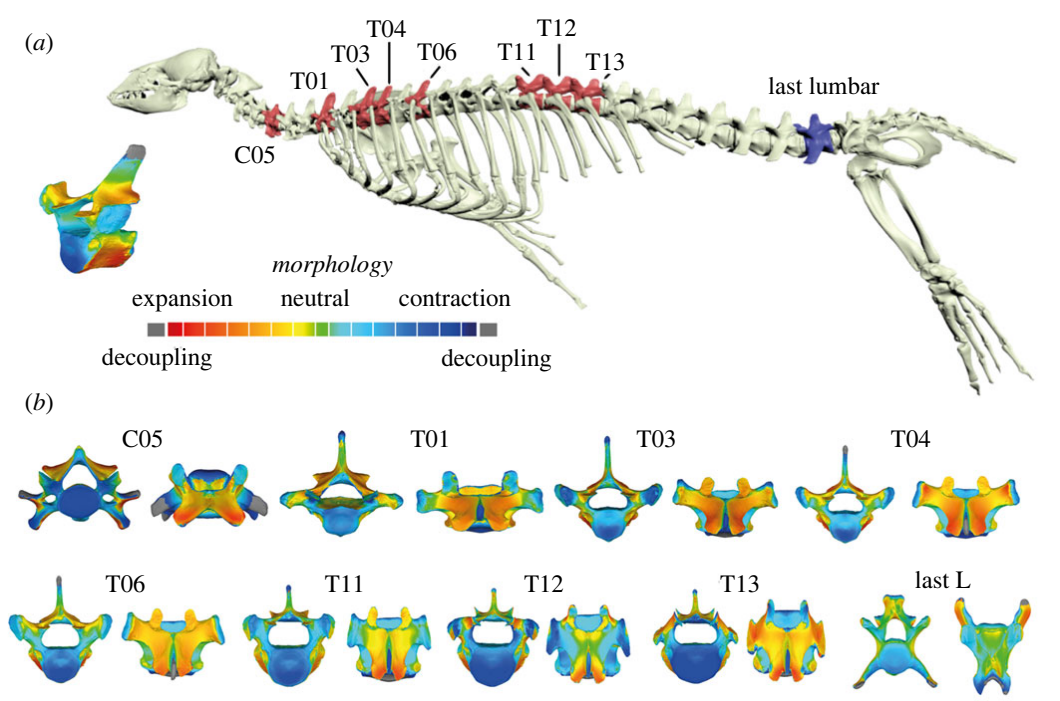
Relationship between vertebral shape and habitat of preference for phocids and otariids. (*a*) Vertebrae with significant ecological signals (electronic supplementary material, table S6) marked in red or purple with the topological deviation analyses of these vertebrae. The morphings were generated using the vertebrae of coastal ecological category as a reference model, and the target model represents the vertebrae of pelagic species. Each vertebra is shown in cranial (left) and dorsal (right) views. See also electronic supplementary material, table S9 for the results obtained from the selected vertebrae approach.

The results of the PGLS analyses per vertebra to test the association between vertebral morphology of living species and habitat of preference (i.e. coastal versus pelagic) yielded significant results for one cervical and seven thoracics when grouping vertebrae by position (figure 5*a,b*; electronic supplemental material, tables S3, S9–13). Therefore, despite the divergence among groups in pinniped axial morphology, species of phocids and otariids that are adapted to pelagic or coastal environments do show shared traits in a cervical vertebra and in some thoracic vertebrae. These changes are related to the shape of the vertebral body, which is more disk-shaped for pelagic species and more cylindrical and narrower for coastal ones. In addition, spinous and transverse processes tend to be shorter for pelagic species in comparison with coastal ones (figure 5*b*). These morphological differences can be attributed to different functional demands imposed by their respective habitats. Disk-shaped vertebral morphology observed in pelagic species enhance vertebral rigidity and enable efficient long-distance movements [15,53,54]. This adaptation is advantageous for pelagic pinnipeds, as their primary mode of locomotion involves swift swimming and covering vast distances. The rigid vertebral joints associated with disk-shaped morphologies contribute to improve propulsion and stability during high-speed cruising [15,53,54]. In contrast, cylindrical vertebral morphologies promote passive flexibility of the joints, and they are associated with pinniped species that are confined to a more coastal environment because they typically engage in agile, high-performance manoeuvrings to pursuit of prey [26].

Strikingly, these vertebral adaptations appear to be independent of the family they belong, which indicates that habitat use influence pinniped vertebral morphology beyond their locomotion mode (pelvic versus pectoral oscillation) [50]. The observed vertebral convergence in pelagic and coastal pinnipeds across otariids and phocids suggests that common ecological influences have shaped their vertebral columns.

Our results agree with the findings of Gillet *et al*. [8] for cetaceans, as there is an association between vertebral shape and habitat. Specifically, riverine and coastal species are distinguished by elongated vertebrae and a lower vertebral count compared to open ocean species.

## 3. Conclusion

By the early Miocene, stem pinnipeds transitioned to freshwater habitats from the traditional terrestrial environment where they adopted a semiaquatic lifestyle [27–29]. This evolutionary transition brought important changes in their vertebral column, probably driven from the partial release of (i) its previous role in body support against gravity and (ii) the fully appendicular locomotion using asymmetrical gaits of fissipeds. Based on our findings, we propose that this liberation facilitated an unconstrained evolutionary scenario leading to the emergence of diverse morphotypes throughout the first two thirds of the pinniped evolutionary history. This scenario differs substantially with the characteristic constrained evolution for the fissiped vertebral column [10]. Moreover, the sequential pattern of increasing disparity from cervicals to lumbars observed in fissipeds [10] is not as evident in pinnipeds (i.e. some thoracics are the most disparate, some lumbars are the less disparate, and the cervicals exhibit intermediate values of disparity).

Our study reveals that the otter-like, stem-pinnipeds, specifically *Pu. darwini* and *Po. valletoni*, possessed vertebral shapes that occupy unexplored areas of morphospaces by crown groups. This is particularly evident in the case of the cervical and lumbar vertebrae. Moreover, when analysing the sequence of the thoracic series, we observe distinct trends that deviate from the established patterns of crown groups. Altogether, these findings highlight the remarkable combination of vertebral shapes exhibited by these early pinnipeds and, hence, highlighting their unique axial skeletons in comparison to their modern counterparts.

On the other hand, disparity through time curves reveal a shift to a scenario of constrained evolution at 10–12 million years ago, a point of time that coincides with a major radiation of crown pinnipeds. The fossil record of odobenids indicates that its peak diversity occurred at 16.0–13.8 Ma (although taphonomic factors cannot be ruled out), but from 13.8 to 11.6 Ma their fossil record decreased substantially [67]. This point of time coincides with a diversity increase of *Allodesmus* (e.g. [68]), probably due to a competitive displacement of desmatophocids over odobenids [67]. However, the diversity of odobenids remains low until the extinction of *Allodesmus* and the diversification of neodobenians [67] at 8–7 Ma.

Although our sample size does not allow to perform DTT analyses on odobenids, vertebral morphologies and disparity-through-time curves in phocids as compared to otariids provide compelling evidence that they followed different paths of axial skeleton evolution. This divergence can be attributed to their highly specialized locomotor strategies, which were firmly established during their respective radiations and subsequent evolutionary time. Phocids rely on pelvic movements to generate thrust and propel themselves through water. In contrast otariids use their pectoral flippers for swimming, resulting in limited utilization of their vertebral columns for propulsion [50].

These differences are also related with other aspects of their ecology such, for example, their feeding behaviour. Phocids use their clawed forelimbs, which play a secondary role in locomotion, to help with the ‘hold and tear’ feeding behaviour [69] whereas otariids develop a different strategy, head shaking in water surface to deal with large prey, as their clawless pectoral flippers are not useful for this task [62,70,71].

We posit that these contrasting locomotor strategies constitute a primary driver for the observed morphological divergences in cervical, thoracic, and lumbar vertebrae across both phocids and otariids, which reinforces the notion of separate evolutionary trajectories for both groups. However, it is noteworthy that despite the variability in locomotor strategies and ecological adaptations of fissipeds, this shape divergence of the spine among crown groups is not observed [7,10]. We hypothesize that the role of the fissiped vertebral column in body support and its contribution in terrestrial locomotion represents a remarkable constraint to evolve towards divergent morphologies.

We also hypothesize that the axial evolution in phocids is subject to greater constraints compared to otariids, because the phocid vertebral column exhibit a rigid anterior region and a highly mobile posterior region, whereas otariids have evolved to maintain maximum flexibility in the entire vertebral column [72]. All vertebrae of phocids, particularly the thoracics and lumbars, are more disparate than the ones of otariids, which probably relates to the role of the vertebral column in swimming, and probably increases morphological variability among different regions depending upon its role in locomotion, such as providing rigidity (the case of posterior thoracics) or mobility (the case of the lumbars). Similarly, all vertebrae of phocids are more constrained that the ones of otariids. The more constrained vertebrae of phocids may reflect that natural selection only allows few morphological solutions but, in the case of otariids, as their vertebral column is only involved in maneuvering and turning, selective pressures are probably more relaxed, and the evolution of vertebral morphology is not as constrained as in the case of phocids.

In any case, our findings strongly suggest divergent pathways within the pinniped axial skeleton, reflecting distinct locomotor adaptations to the challenges posed by the marine environment. Therefore, we hypothesize that the unique locomotor strategy deployed by each pinniped family played a pivotal role in shaping their respective regional morphologies. However, our ecomorphological analyses also reveal shared traits between coastal and pelagic species within phocids and otariids. In fact, we have identified a significant influence of preferred habitat on the morphology of several thoracic vertebrae, regardless of the family that taxa belong and of the locomotor strategy they deploy. Interestingly, despite the clear differentiation between vertebral morphologies of phocids and otariids given their distinct locomotory modes (pectoral rowing versus pelvic oscillation), individual vertebrae of coastal or pelagic species from both groups share similar morphologies.

## Data Availability

The data are provided in electronic supplementary material [73].
